# Specificity ratings for English data

**DOI:** 10.1007/s10339-024-01239-4

**Published:** 2024-11-08

**Authors:** Andrea Amelio Ravelli, Marianna Marcella Bolognesi, Tommaso Caselli

**Affiliations:** 1 ABSTRACTION Research Group, Department of Modern Languages, Literatures and Cultures, University of Bologna, Via Cartoleria 5, 40124 Bologna, BO Italy; 2 Center for Language and Cognition, Faculty of Arts, University of Groeningen, Oude Kijk in ’t Jatstraat 26, 9712 EK Groningen, The Netherlands

**Keywords:** Abstraction, Categorization, Human ratings, Specificity, Concreteness, Best–worst-scaling, Cross-language comparison

## Abstract

A dataset of specificity ratings for English words is hereby presented, analyzed and discussed in relation with other collections of speaker-generated ratings, including concreteness. Both, specificity and concreteness are analyzed in their ability to explain decision latencies in lexical and semantic tasks, showing important individual contributions. Specificity ratings are collected through best–worst scaling method on the words included in the ANEW dataset (Bradley and Lang in Affective norms for English words (ANEW): instruction manual and affective ratings (Tech. Rep.). Technical report C-1, the center for research in psychophysiology, 1999), chosen for its compatibility with many other collections of rating resources, and for its comparability with Italian specificity data (Bolognesi and Caselli in Behav Res Methods 55(7):3531–3548, 2023), allowing for cross-linguistic comparisons. Results suggest that specificity plays an important role in word processing and the importance of taking specificity into consideration when investigating concreteness effects.

## Introduction

Abstraction, a hallmark of human cognition, remains a rather vague phenomenon that possesses intuitive comprehensibility, yet eludes precise delineation. A recent definition offers a potential explication of Abstraction as “The process of forming general ideas or concepts by extracting similarities and general tendencies from direct experience, language, or other concepts” (Reilly et al. 2024). Upon a closer examination of this overarching definition, identifying the fundamental mechanisms involved in this process proves to be challenging. One of the difficulties arises from the tendency for definitions of Abstraction to often confound two distinct variables, which can be identified as Concreteness and Specificity (for a discussion on this matter, see Bolognesi et al. 2020; Bolognesi and Caselli 2023).

Concreteness is commonly defined as the extent to which the referent designated by a linguistic term can be perceived through sensory experiences (Brysbaert et al. 2014). In this context, a term such as *banana* designates a referent that can be seen, touched, smelled, tasted and therefore it is commonly associated with a high score of concreteness. Conversely, a term like *belief* is commonly associated with a low score of concreteness.

Specificity can be defined as the level of inclusiveness or precision of a conceptual category (see Iliev and Axelrod 2017; Bolognesi et al. 2020; Bolognesi and Caselli 2023): a word like *banana* defines a quite precise and limited category that includes members that share many common features (e.g., *baby bananas*, *long bananas*, *rotten bananas* among others) while a category like *food* is quite generic and highly inclusive, because it includes various category members that do not necessarily share many common features (e.g., *bananas*, *soups*, *popcorn*).

Recent studies indicate a positive correlation between these two variables when Concreteness is operationalized through concreteness ratings obtained from human respondents using Likert scales (e.g., Iliev and Axelrod 2017; Bolognesi et al. 2020) and Specificity is operationalized through scores extracted from WordNet (Miller et al. 1990; Fellbaum 1998), an electronic lexical resource in which words are organized hierarchically and linked by relations of semantic inclusion. WordNet, unlike human ratings, has been constructed by lexicographers with the support of external knowledge bases and dictionaries. Iliev and Axelrod (2017), in particular, report that the effects of Concreteness and Specificity on decision latencies diverge significantly: higher specificity leads to prolonged processing times in lexical decision tasks, while higher concreteness leads to shorter processing time, aligning with the so-called “concreteness effect” (Hajibayova 2013). In both these studies, the authors acknowledge the limitations in using WordNet as a source for the operationalization of categorical Specificity. In a more recent study, Bolognesi and Caselli (2023) build on the limitations outlined above and collect Specificity ratings for a dataset of roughly one thousand words in Italian, for which other psycholinguistic norms are available. The dataset consists of the ANEW-IT norms (Montefinese et al. 2014), which is the Italian version of the Affective Norms for English Words (ANEW) dataset (Bradley and Lang 1999). For this dataset, various normed data are available in Italian, including Affective scores, Age of Acquisition scores, Familiarity scores, Concreteness scores, Imageability scores, as well as Decision Latencies collected in Lexical Decision and Naming Tasks (Vergallito et al. 2020). The method used to collect Specificity ratings is the Best–Worst Scaling method (BWS) (Flynn and Marley 2014), which is described in further detail in the section “Theoretical background”. The basic idea is that the partial ranking involved in the Best–Worst mechanism allows users to relate word meanings to one another rather than judging a semantic dimension of their meaning in absolute terms, like in rating tasks. As a matter of fact, in a Best–Worst task, users are invited to indicate the best and the worst item within a group of items, in relation to a variable of interest. In this case: Specificity. Given a group of words, users shall indicate the most specific and the least specific (i.e., most generic). Given that Specificity is a relational property of word meaning, as argued by the authors, this method seems more appropriate to collect Specificity scores, compared to a classic rating task on Likert scales. Bolognesi and Caselli (2023) report that word Specificity is positively correlated to word Concreteness as well as Imageability and Age of Acquisition, while it is negatively correlated with Frequency, Familiarity and Affective content. Moreover, Specificity explains a significant fraction of Decision Latencies in Lexical Decision Tasks, above and beyond other psycholinguistic variables, commonly acknowledged to affect word processing. Finally, a quadratic fit gives even better results for Specificity, suggesting that words belonging to the basic level of Abstraction tend to be recognized faster, compared to words belonging to the superordinate level (therefore generic words) and to the subordinate level (therefore, highly specific words), as argued also by previous research (Rosch et al. 1976).

The partially converging evidence provided by Iliev and Axelrod (2017) who used WordNet to operationalize Specificity on a dataset of English words and Bolognesi and Caselli (2023) who operationalized Specificity using crowdsourced judgments on a dataset of Italian data, suggests that Specificity and Concreteness are two theoretically distinct variables that impact word processing in different ways. This is a particularly crucial finding, considering that most studies focused on language processing and on the distinction between concrete and abstract words and concepts do not control for Specificity because of a general lack of lexical resources that can be used to operationalize this variable. Within this state of the art, the aims of the present article can be summarized as follows: (1) to provide a dataset of Specificity ratings collected from human judges using the Best–Worst scaling method on English data, (2) to provide an in-depth analysis of how Specificity relates to Concreteness as well as to other psycholinguistic variables that are often involved in the study of Abstraction, looking at English data; (3) to explore and discuss potential cross-linguistic differences between English and Italian and (4) to discuss how the findings of our analyses relate to the general mechanism of Abstraction and semantic categorization.

We therefore address the following research questions in the coming sections:

- RQ1: What’s the relation between Specificity and Concreteness as well as between Specificity and other psycholinguistic variables commonly involved with the study of Abstraction? ("Results: What’s the relation between specificity and concreteness as well as between specificity and other psycholinguistic variables commonly involved with the study of abstraction?" section)
- RQ2: How does Specificity impact word processing alone, in interaction with Concreteness, and when other psycholinguistic variables are controlled? ("Results: How does specificity impact word processing alone, in interaction with concreteness, and when other psycholinguistic variables are controlled?" section)
- RQ3: Does Specificity change across languages and how? ("Results: Does specificity change across languages and how?" section)

We illustrate and motivate our hypotheses in the section “Theoretical background”.

## Theoretical background

In cognitive linguistics, psycholinguistics, cognitive science and psychology a longstanding tradition focuses on the understanding of how people acquire, process and produce abstract versus concrete words and concepts (e.g., Hoffman 2016).

The concreteness effect, as mentioned in "Introduction" section, denotes an advantage that concrete words have over abstract words in various tasks, like reading, processing, recalling, naming, and recognizing (e.g., Borghi et al. 2017; Villani et al. 2019). Empirical evidence about the “concreteness effect” piled up in the past decades, while the origins of such effect and how it works in the human brain have been subjects of lively debates (e.g., Vigliocco et al. 2011). A component that muddies the waters of this theoretical debate is that empirical evidence supporting the concreteness effect is typically built on experiments based on stimuli that have been selected based on concreteness ratings, which are human judgments provided on Likert scales, about the perceived concreteness of given prompts (e.g., Brysbaert et al. 2014). Concreteness ratings have been recently criticized for their ambiguity. For instance, Reijnierse et al. (2019) show that such ratings are sometimes attributed to words that are inherently polysemic (e.g., *field*) in that they encompass both, a literal meaning, which is usually quite concrete (e.g., a *field* of tomatoes) and a metaphorical meaning which is often abstract (e.g., a *field* of research). When these two meanings are disambiguated to participants, very different concreteness scores are produced. In a more recent work Montefinese and colleagues (Montefinese et al. 2023; Gregori et al. 2020) release a dataset containing values of concreteness scores provided in context, showing that Concreteness for the same word can vary consistently, depending on the context of occurrence and the sense that contexts activate.

The unresolved nature of Concreteness ratings led to the question of what people judge and measure when they are asked to score the concreteness of a concept. In trying to unpack the information encoded in Concreteness ratings, scholars advanced different hypotheses and collected different alternative ratings. For instance, scholars have collected modality-specific norms of perceptual strength measuring how much each of the five senses contributes to construct a conceptual representation (namely: to what extent is a concept related to vision, touch, hearing, olfaction and taste). Research shows that these norms better reflect the actual perceptual content of words and concepts, and predict decision latencies with a slightly higher accuracy, compared to concreteness ratings which, in turn appear to be biased toward the visual modality and appear to condense more information than simple perceptual content (Connell and Lynott 2012). Other approaches have attempted to unpack concreteness ratings by collecting ratings on new variables, and showing to what extent such variables could explain the variance in concreteness ratings. For instance, Troche et al. (2017) hypothesize that a range of cognitive and perceptual dimensions (e.g., emotion, time, space, color, size, visual form) contribute to forming a conceptual topography that can explain variability in Concreteness. Davis and Yee (2023) show that some 50% of the variance in Concreteness ratings is explained by how much time and how much physical space is required to perceive a concept. While the experimental unpacking of the notion of Concreteness is still an ongoing objective in cognitive science and related disciplines, with researchers trying to identify new variables that can contribute to explain the concreteness effect, from a theoretical standpoint we argue that the variable of categorical Specificity is a well-known but neglected one in many disciplines, including linguistics and cognitive science. The level of semantic categorization at which a word labels a group of referents has shown to have an impact on chronometric data (Lexical Decision Tasks), and such impact is, in some cases, higher than the impact of Concreteness (Bolognesi and Caselli 2023).

In the present paper, we investigate how the categorical Specificity of English words relates to other psycholinguistic variables that are commonly acknowledged to impact word processing, and -in particular- how Specificity relates to Concreteness. In a first set of analyses ("Results: What’s the relation between specificity and concreteness as well as between specificity and other psycholinguistic variables commonly involved with the study of abstraction?" section) we introduce the dataset of Specificity ratings collected for roughly one thousand English words, and: (1) we compare them with the Specificity scores that can be automatically extracted from WordNet to assess their plausibility; (2) we relate them with Concreteness ratings as well as with other psycholinguistic variables commonly associated with the notion of Concreteness. In this study, the aim is therefore to understand how Specificity is positioned in relation to Concreteness and other psycholinguistic variables, in the attempt of contributing to unpack the unresolved notion of Concreteness. We expect to find a positive, medium correlation between the specificity ratings collected on English language and the specificity scores extracted from WordNet, as previous research based on Italian language shows (Bolognesi and Caselli 2023). Moreover, we expect to find a similar pattern of correlations already observed for Italian: positive correlation with word Concreteness, Imageability and Age of Acquisition, and negative correlation with Frequency, Familiarity and Affective content. In addition, we ran regressions to investigate the role of Specificity in explaining Concreteness ratings, when other variables are considered. If the positive correlation between Specificity and Concreteness is supported, then we argue that the facilitatory effect commonly attributed to word Concreteness and labelled as the “concreteness effect” could possibly be explained also by Specificity, a variable that is typically neglected in behavioral studies because of the lack of resources that could be used to operationalize it.

In the second set of analyses ("Results: How does specificity impact word processing alone, in interaction with concreteness, and when other psycholinguistic variables are controlled?" section) we examine the notion of Specificity and investigate its role in explaining decision latencies (chronometric data), alone and in interaction with Concreteness. We expect to find that Specificity alone explains decision latencies and in interaction with Concreteness it may drive the effect over such latencies. Moreover, we expect to observe that Specificity explains a fraction of decision latencies above and beyond Concreteness and other psycholinguistic variables, supporting the idea that Specificity is a theoretically distinct variable, compared to Concreteness and given its role in semantic access, it should be carefully taken into consideration. Hence, our dataset of Specificity ratings, which can help researchers to operationalize and measure this variable.

Finally, in a last set of explorative analyses ("Does specificity change across languages and how?" section) we compare English Specificity ratings with Italian Specificity ratings collected with the same methodology (i.e., Best–Worst scaling) and on the Italian translation of the same set of stimuli by Bolognesi and Caselli (2023), discussing similarities and differences between the two languages. We complete the cross-lingual analysis by extending the comparison to other psycholinguistic/semantic features collected in both languages with rating scales. We expect overall to find similarities about the role of Specificity in language processing and in explaining decision latencies. Moreover, comparing the specificity ratings obtained with the Best–Worst scaling method, we might expect to see systematic differences between English and Italian data, in the ways the scales are reconstructed from the Best–Worst judgments. In fact, based on classic ratings scales, previous research (Van Herk et al. 2004), has suggested that Italians systematically provide higher ratings, compared to other populations. It will be interesting to see whether this tendency can be observed in ratings collected using a different method, in our case the Best–Worst scaling.

## Methods

### Collection of specificity ratings

In line with Bolognesi and Caselli (2023), the present collection of Specificity judgments is based on the words included in the ANEW dataset (Bradley and Lang 1999). The choice of this dataset is driven by two main motivations: on one hand, the possibility of comparing the aspect of Specificity inter-linguistically between the already collected Italian ratings and the English ratings that are the object of this study; on the other, the possibility to analyze the interplay of Specificity with a series of other variables which have been previously collected about these words.

We applied the methodology described by Kiritchenko and Mohammad (2017), and we used their scripts to create the tuples, to analyse the split-half reliability and to compute the Best Worst Scaling.^1^

Starting with the words from the 1034 words of the original ANEW dataset, we built 4-words tuples. Tuples are generated by random sampling from the pool of target words, and according to the following criteria:

- no two words within a tuple are identical;
- each target word appears approximately in the same number of tuples;
- each pair of words appears approximately in the same number of tuples.

We kept the target words separated by Part-of-Speech before creating the tuples, to avoid introducing bias. This resulted in annotations tasks focused either on nouns-only, verbs-only or adjectives-only tuples. Moreover, some words have the same morphosyntactic form for more than one Part-of-Speech, and thus it may be difficult to disambiguate them if presented in isolation. For this reason, words such as *yellow*, *fat*, *adult*, and so on, which can be both nouns and adjectives depending on the context in which they are used, have been used to build mixed POS tuples and they have been presented in separate lists.

This process led to 2,068 4-words tuples, which have been distributed in 53 questionnaires with maximum 40 tuples each, plus 2 attention check tuples. For each list, we collected at least 10 valid annotations. To limit the possibility of collecting too many annotations from one single rater (i.e., one person submitting judgments to multiple questionnaires), we delayed the distribution of the questionnaires by publishing them in batches of about 10 questionnaires at a time on a crowdsourcing platform, and by inhibiting the participation of raters from old batches on the new ones. Indeed, very few raters completed more than one questionnaire: only five participants rated a total of 3 questionnaires, and 26 worked on 2. In other words, the majority of the participants expressed their judgements on 40 target stimuli, 26 of them on 80 stimuli, and 5 of them on 120.

**Fig. 1:**
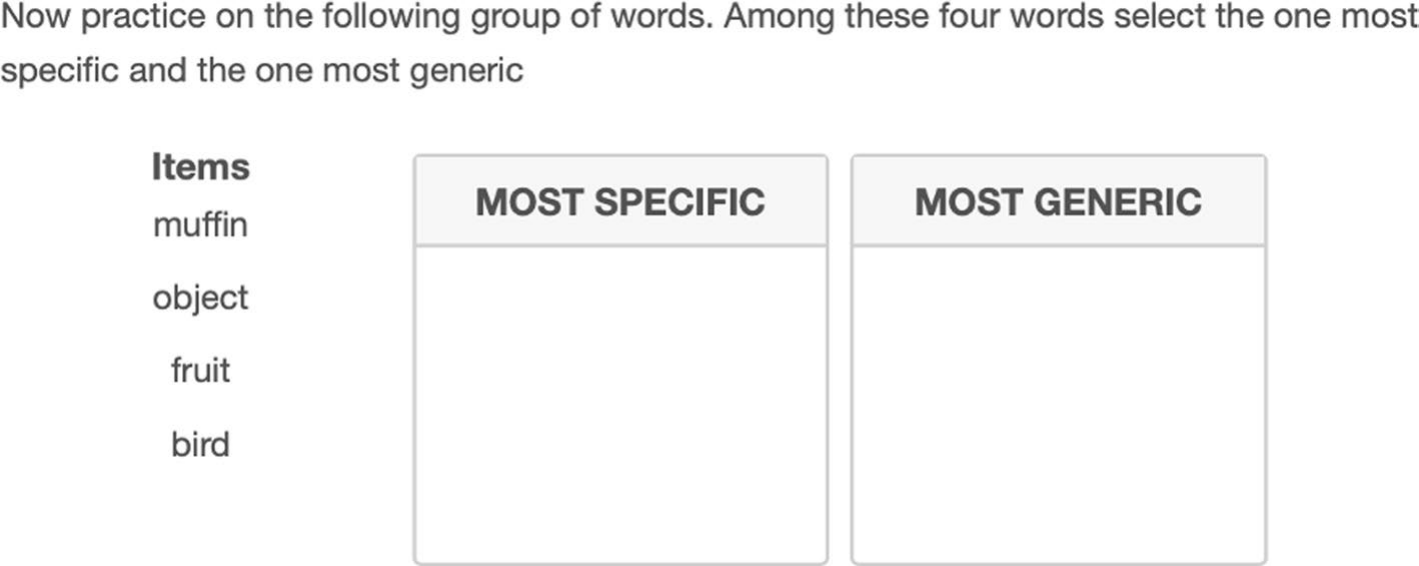
Trial tuple used to verify the comprehension of the task instructions

The rating task, in the form of a survey, has been designed to be completed in about 20 min. After an introduction to the research’s scope, raters were presented with the informed consent and privacy policy. We then asked for some sociodemographic information while preserving anonymity (age, gender, and education), and proceeded with giving instructions about the task. We then inserted a trial 4-words tuple for practice, that had the same design as the experimental trials, displayed in Fig. 1.

The practice trial was the only section of the survey where participants received automatic feedback (i.e., an error message) on their choices, when they did not select the most specific (*muffin*) and the most generic (*object*).

Through the list of experimental trials, we added two control trials (i.e., attention checks) to judge whether the participant was going through the task with the necessary effort to ensure a reliable set of judgments. As control trials we used tuples containing words that refer to concepts in a taxonomic relation, i.e., all chained with a hyponym/hypernym relation. The idea is that, beyond subjectivity, a taxonomic series may not pose any doubt on the individuation of the most generic and most specific words, thus going wrong on these can be interpreted as a signal of a lack of attention and/or effort in completing the task.

The verification of the attention checks has been automatic but not *strict*, in the sense that we did not consider absolute right choices, but rather we looked at the Specificity/Genericity relative to the two words the participant selected. Given the taxonomic relation, the verification has been easily conducted by assigning to each word in the tuple a value in the 1–4 range (1 = most generic; 4 = most specific) and then we automatically checked that the selected most specific word had a higher value than the one selected as most generic. If a participant failed at least one of these two checks, we excluded him/her from the study.

### Participants

Participants were recruited through the Prolific crowdsourcing platform^2^ (Palan and Schitter 2018), and the task was set up on Qualtrics.^3^ In total, 531 English native speakers took part to our study. We used screeners provided by the platform to ensure that participants were English native speakers, born and raised in an English-speaking country (i.e. a country for which English is the official first language), and not random crowdsourcing workers with just a good fluency in English. All the materials, including the set of screeners, the task instructions and the analyses, are stored in the online repository: https://osf.io/zm283/?view_only=8d5ff8bdb27b4bb8b4007ee8b0208686.

We collected data from an evenly distributed sample of adult English speakers. The mean age of the participants is 42.39, with a standard deviation of 14.44. The sample is balanced by sex, with about the half of the participants having a Bachelor’s Degree, and more than three-quarters of the participants are born in the United Kingdom, where they also spent most of their childhood and where they still reside. About 10% of the participants are enrolled in an education program (i.e. they identified themselves as *students*).

### Data cleaning and preparation

The annotation campaign involved a total of 531 participants, from whom we obtained 560 complete annotation sessions. We excluded 39 of these sessions due to failure in attention checks. The exclusions represent only the 6.96% of the collected judgments, and we can see this both as a confirmation of the feasibility of the annotation of Specificity through BWS methodology, as well as a demonstration of good comprehension of the instructions given to the participants.

At the end of the collection campaign, and after the exclusion of unreliable raters, we verified the reliability of the data by means of an average split-half reliability (SHR) test over 100 trials, as proposed by Kiritchenko and Mohammad (2017). We run the reliability test on the whole set of annotations, on the POS-specific set of lists, and on single annotation lists.

**Table 1:** Split-half reliability (avg. on 100 runs) for the specificity ratings, divided per part-of-speech and in total

Set of stimuli	Pearson (std)	Spearman (std)
Nouns-only	0.96 (0)	0.96 (0)
Verbs-only	0.91 (0.02)	0.88 (0.02)
Adjectives-only	0.9 (0.01)	0.89 (0.01)
All POS	0.95 (0)	0.95 (0)

In brackets, standard deviation

Table 1 shows the results for POS-specific lists and on the whole set of annotations (All POS), while the detailed results of the SHR tests on single annotation lists can be found in the OSF repository. The resulting correlation is extremely high (0.95 with both Pearson and Spearman on the whole set of ratings), with nouns-only ratings leading the ranking, but also on the other parts-of-speech we can see a convincingly high correlation. These results confirm a good reliability and reproducibility of the collection, and they are also in line with the reliability of the specificity ratings collected for the Italian language on the translated version of the ANEW, as it has been conducted by Bolognesi and Caselli (2023).

## Results: What’s the relation between specificity and concreteness as well as between specificity and other psycholinguistic variables commonly involved with the study of abstraction?

### Comparison between crowdsourced specificity (BWS) and expert-compiled (WordNet) specificity

**Table 2:** Comparison of descriptive statistics between specificity values obtained with BWS-crowdsourcing and WordNet

	BWS specificity	WordNet specificity
Total words	669	
Mean specificity	0.548	0.322
STD specificity	0.227	0.162
25% (quartile 1)	0.364	0.187
50% (quartile 2)	0.554	0.312
75% (quartile 3)	0.728	0.437

**Fig. 2:**
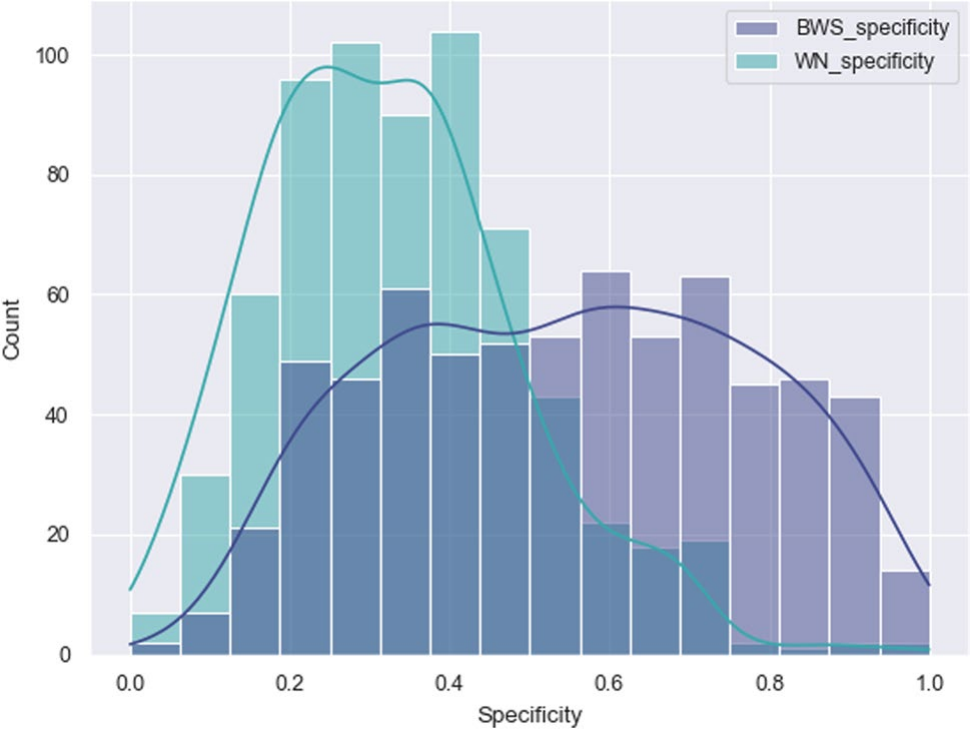
Frequency distribution of the specificity scores obtained with BWS and the specificity scores extracted from WordNet

**Fig. 3:**
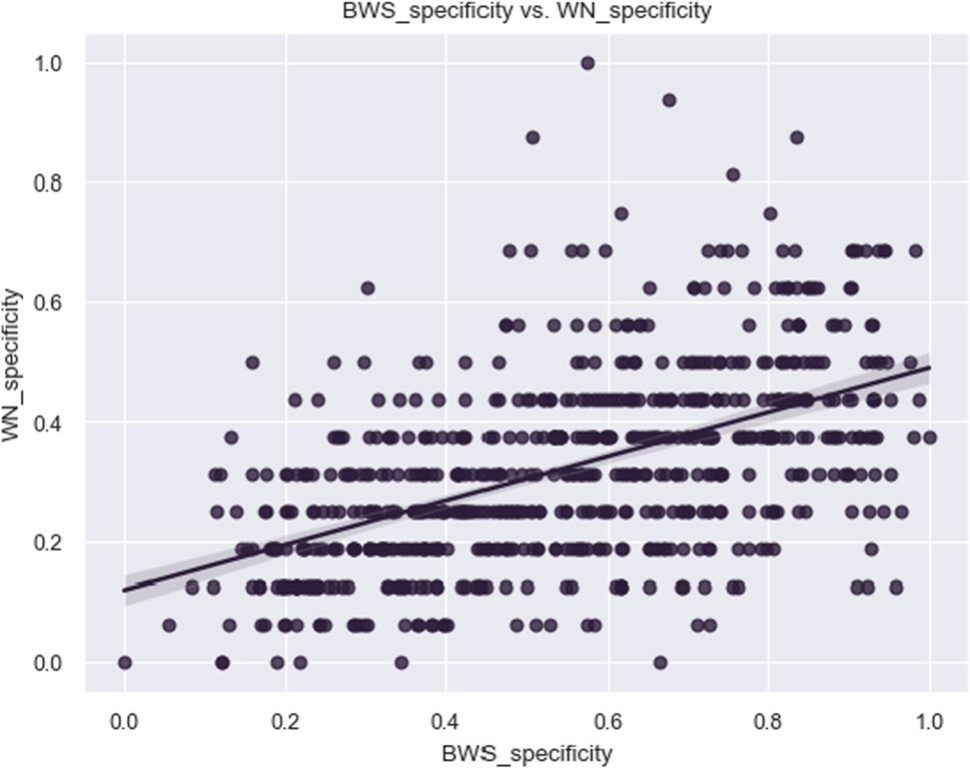
Plot of correlation between specificity obtained from WordNet and from BWS

We conducted an initial analysis by comparing specificity data obtained through the BWS methodology (crowdsourced ratings) with specificity scores derived from WordNet (Miller 1995; Fellbaum 1999). To extract Specificity scores from WordNet, we used the “Specificity 3” metric described in Bolognesi et al. (2020), which takes into account the overall depth of the WordNet taxonomy and the relative position of a word therein, using the following formula:

$$\begin{aligned} WNspecificity = (1 + d) / 20 \end{aligned}$$

where *d* is the distance of a node to the root node of the taxonomy, and 20 is the maximum depth of WordNet.

We applied this method to nouns exclusively, and thus the comparison is made on 669 (66% of the total stimuli), that is the number of the nouns shared between the ANEW set and the WordNet taxonomy. To make the data from both sources comparable, we scaled the values in the 0–1 range with the MinMaxScaler method from the ScikitLearn Python Library (Pedregosa et al. 2011).

Figure 2 shows the distribution of Specificity ratings in the two metrics (BWS and WordNet). WordNet scores are skewed toward the left (i.e. they register overall lower Specificity) while BWS data is distributed more evenly along the whole scale (see below for normality test). This trend is confirmed also if we look at Table 2, where mean values and quartile distribution of entries are reported. While the mean value of the specificity from BWS falls in the middle of the 0–1 range of specificity, the mean from WordNet is strongly shifted downward, with a difference of about 20% with respect to the first. This is also confirmed from the quartile distribution of the datapoints: more than the 70% of the datapoints from WordNet falls in the lower half of the Specificity scale.

Given that neither of the two datasets is normally distributed, we measured the divergence of the two with a Mann–Whitney U test, which confirmed that the two distributions are different (p value significantly lower than 0.05). Still, the two distributions show a moderate correlation (Spearman coefficient: 0.540, $p\,value < 0.05$), as it can be observed in Fig. 3.

### The relation between specificity and other psycholinguistic variables, including concreteness

**Table 3:** List of psycholinguistic variables gathered for comparison with specificity

Variable	Original collection
Concreteness	Concreteness norms (Brysbaert et al. 2014)
Valence	XANEW (Warriner et al. 2013)
Arousal	
Dominance	
Imageability	Glasgow norms (Scott et al. 2019)
Familiarity	
Age of acquisition	
Frequency	ukWaC frequency list (Baroni et al. 2009)

Table 3 reports all the psycholinguistic variables used for these analyses, along with the dataset they are taken from. We initially compared Specificity and Concreteness using ratings derived from Concreteness norms (Brysbaert et al. 2014). We then explored interactions with Valence, Arousal, and Dominance, aligning with the three-dimensional theory of emotions (Osgood et al. 1957). Valence denotes emotional polarity (i.e., happy vs. unhappy, with neutral in the middle), Arousal measures the intensity of the emotional activation, and Dominance gauges perceived control over a stimulus. These emotional features were sourced from Warriner et al. (2013).

Additionally, we considered Imageability, Familiarity, and Age of Acquisition as descriptors of a word’s relevance in daily life. Imageability is the measure of the cognitive effort required to create a mental representation, i.e., a mental image, of the concept evoked by a word. This semantic dimension has been shown to be in high correlation with Concreteness (Paivio et al. 1968): the more concrete the concept evoked by a word, the more imageable it is. Moreover, the processing of highly imageable words is faster and easier with respect to less imageable words (Balota et al. 2004; Cortese and Schock 2013). Familiarity is the measure of how much a word is commonly used, heard, or read daily, thus it records how much a word (more precisely, the concepts behind that word) is part of the individual speaker’s experience of the everyday world. Age of Acquisition refers to the age at which a word is presumably acquired at first. This measure has been demonstrated to impact performance across numerous cognitive tasks (Johnston and Barry 2006): typically, words learned earlier in life are recognized faster than those acquired later. Finally, we considered the Frequency of the words as an objective measure in our comparison. We used the list of lemmas with frequency distribution from ukWaC, i.e. the English section of the Wacky Corpora (Baroni et al. 2009). Given that Wacky Corpora are derived from automatic scraping and cleaning of pages from the web, we cleaned the dictionary by filtering out all those lemmas that are not present in the repertoires for the English language from the Universal Dependencies project (Nivre et al. 2017), which are mostly manually curated and validated. We applied a logarithmic transformation to raw frequencies in order to mitigate the typical skewness of the frequency distributions: few words are absolutely frequency, with a long tail of rare words (and hapaxes, in some cases). We decided to consider word frequency distributions computed over texts and documents from the web due to the increasing use of digital platforms for written language consumption. In this way, we can rely on a more ecological and representative approach when considering possible effects of Frequency over Specificity. Similarly to the comparison between BWS and WordNet Specificity, all variables have been scaled in the 0-1 range using the MinMaxScaler method to avoid possible magnitude effect on the subsequent analysis, and to make them easily comparable.

**Fig. 4:**
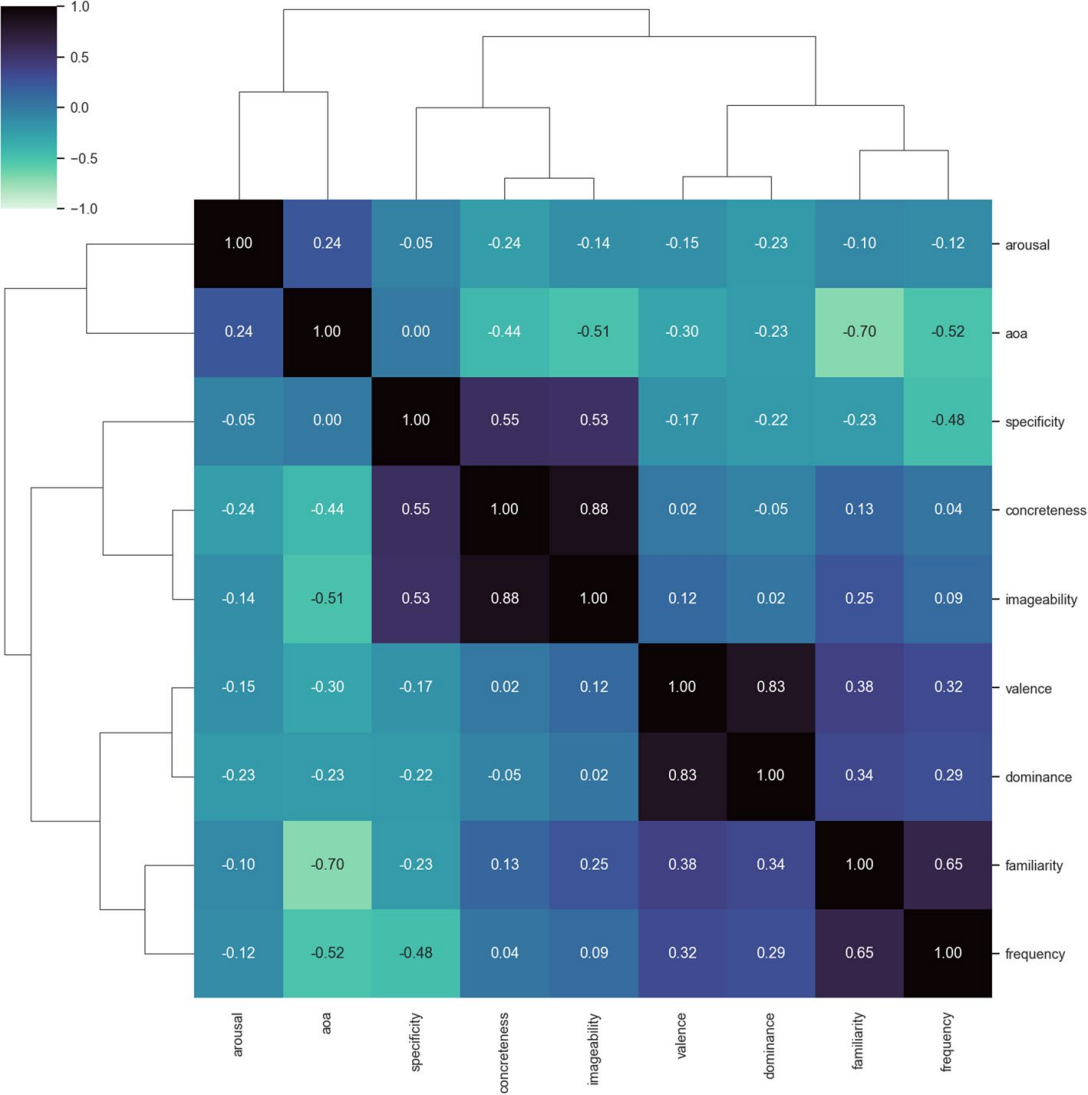
Cluster map of Spearman correlations among all the features

As shown in Fig. 4, it is clear that some variables are higly correlated, such as Valence and Dominance (Spearman rho: 0.833), and Concreteness and Imageability (Spearman rho: 0.88), thus it is possible to hypothesize a collinearity effect among the collected variables. To confirm this, we calculated the Variance Inflation Factor (VIF) for all variables, which indicated that Imageability had the highest score (VIF 5.784), signaling a significant but not strong collinearity in the data due to its presence. Upon removing Imageability and recalculating VIF values, we obtained results with VIFs below the cut-off of 4 (Max: 3.94, mean: 2.553). As a result, we decided not to consider Imageability in our subsequent analysis.

**Fig. 5:**
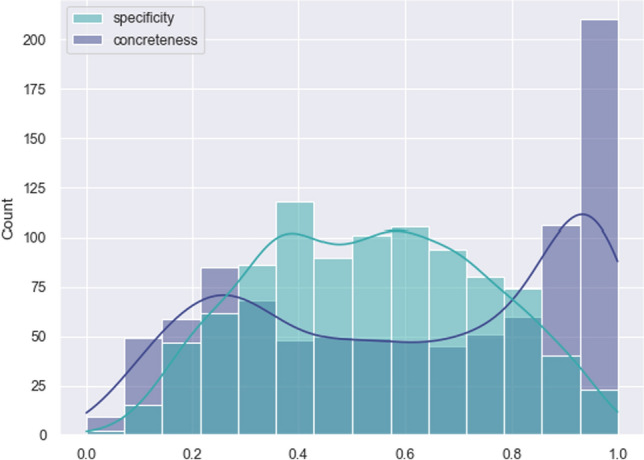
Frequency distribution of specificity and concreteness scores on the ANEW words

**Fig. 6:**
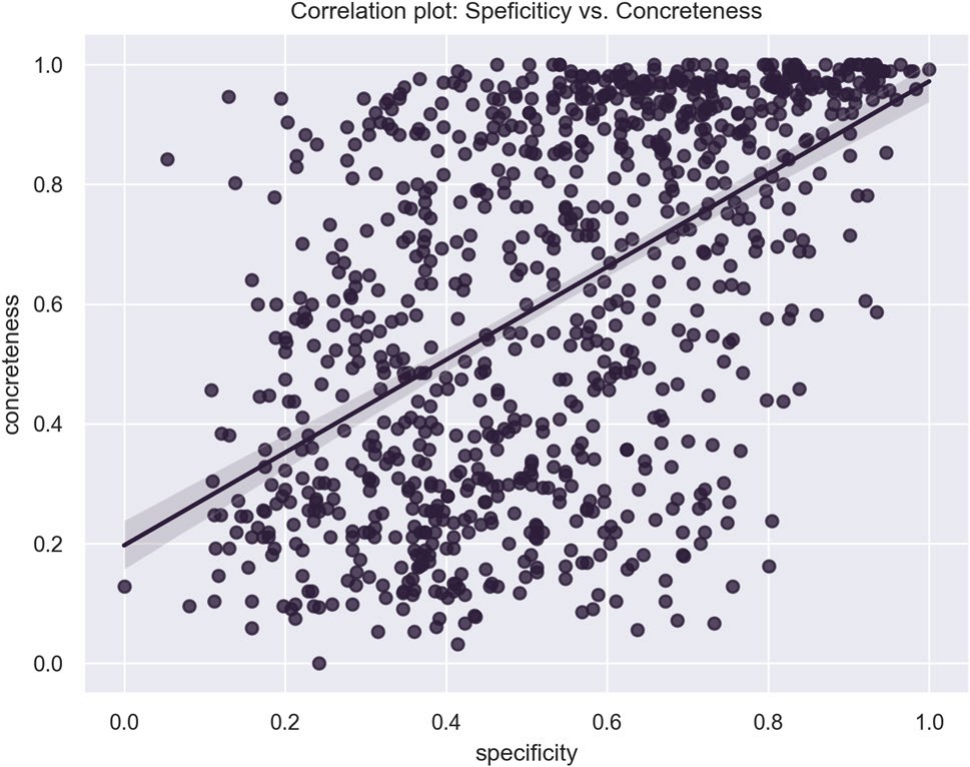
Plot of the correlation between specificity and concreteness on the words of the ANEW dataset

Specificity and Concreteness in the ANEW set of words are not normally distributed and highly divergent (Fig. 5). A Mann–Whitney test, used to assess whether there is a significant difference between two independent datasets, showed that this is the case ($p < 0.05$). A correlation analysis showed that, mostly, the higher the Concreteness, the higher the specificity, with a Spearman rho of 0.552 ($p\, value < 0.05$), as showed in Fig. 6.

**Table 4:** Report of correlations and regressions of specificity against the listed variables

Variable	Spearman rho	r-squared
Concreteness	0.552*	0.291
Valence	$-$0.173*	0.042 *(q)*
Arousal	$-$0.049	0.001
Dominance	$-$0.216*	0.055 *(q)*
Familiarity	$-$0.228*	0.069 *(q)*
Age of acquisition	8.8872e$-$06	0.000
Frequency	$-$0.475*	0.197 *(q)*

Asterisks in the Spearman rho column indicate a $p \, value > 0.01$; *q* in the r-squared column stands for quadratic regressions

In relation to other metrics, Table 4 summarizes the analysis of the correlation and regression between Specificity and the other psycholinguistic variables described above. The results of the correlation indicate that Specificity shows a moderate negative correlation with Frequency, and a low negative correlation with Valence, Dominance, and Familiarity. Age of Acquisition (AoA) and Arousal do not attain statistical significance in this context. Moreover, we tested the predictive power of Specificity over the psycholinguistic variables by performing both linear and quadratic Generalized Least Square regressions, and we selected the model that best minimize the Bayesian Information Criterion (BIC). As expected, the regressions show a trend that is coherent with the correlations: Specificity alone is able to explain about the 29% of the variation on Concreteness, and a bit under the 20% on Frequency, while its predictive power is not relevant for the other variables, and it has no significance for Arousal and Age of Acquisition.

### Ad interim discussion

Our study’s results shed light on the relationships between Specificity and Concreteness, and between Specificity and other psycholinguistic variables commonly associated with the study of abstraction mechanisms.

We reported a substantial positive correlation between the Specificity ratings collected from human judgements (BWS) and those extracted from WordNet, in line with our hypotheses. The correlation however is not high, suggesting that speakers’ perception of categorical Specificity and taxonomic Specificity encoded in WordNet are meaningfully associated but also theoretically distinct. For instance, generic abstract concepts such as *chance* (BWS: 0.158; WN: 0.188), *opinion* (BWS: 0.184; WN: 0.188), or *quality* (BWS: 0.120; WN: 0), generic concrete ones, such as *food* (BWS: 0.129; WN: 0.062), *body* (BWS: 0.195; WN: 0.125), or *item* (BWS: 0.053; WN: 0.062) and specific concrete concepts such as *tobacco* (BWS: 0.803; WN: 0.750), *dog* (BWS: 0.818; WN: 0.688), or *horse* (BWS: 0.834; WN: 0.688), they all behave in similar ways when we look at crowd-sourced Specificity and expert-compiled Specificity. Conversely, for specific abstract concepts the situation is more complicated, and to find some examples of abstract concepts for which both BWS and WN agree on the assessment of Specificity, we must consider a wider gap between the two measures, and also concepts placed more closer to the center of the Specificity distribution and not absolutely abstract, such as *snob* (BWS: 0.520; WN: 0.438).

Moreover, the two measures of Specificity assign very diverging scores in some cases, such as for the word *radiator* (BWS: 0.959; WN: 0.125), *crown* (BWS: 0.908; WN: 0.125), and *rainbow* (BWS: 0.902; WN: 0.250). It is worth noting that the cases with greater discrepancy can be found in the higher portion of the Specificity scale collected using the BSW method, and much more concrete. Probably, many of these divergent cases face the same problems observed by Bolognesi et al. (2020), and connected both to the internal structure of the WordNet taxonomy and to the Specificity computing methodology implemented. On one hand, WordNet is a collection of SynSets (i.e., word senses), and for this reason querying for a word consists of selecting all the SynSet in which the requested word appears. The sorting of these SynSet is not controlled: they are always listed on the basis of the part-of-speech to which the sense refers, but a part of this there is no specific rule for sorting. This and the level of granularity of the resource make it difficult to select the most appropriate sense to be used for computing the Specificity score. On the other hand, the implemented method considers exclusively the first listed sense for the searched word, so the value of Specificity is always that of the first sense in the taxonomy. Nevertheless, disambiguation without any context to anchor the meaning is impossible. As Montefinese et al. (2023) observed, the level of Concreteness of words in context (i.e., the stimulus is composed of a phrase in which the target word appears) is more stable, in terms of lower standard deviation, for words with only one SynSet in WordNet, while greater variability is observed for words connected to multiple SynSets.

Subsequently, we reported a substantial positive correlation (Spearman rho: 0.552) between Specificity and Concreteness ratings. This indicates that as the Concreteness of a word increases, its Specificity also rises. Essentially, more concrete words tend to be more specific. However, also in this case, the non-perfect association suggests that the two variables are different. As a matter of fact, one could find words that are specific and concrete, such as *maggot* (Specificity: 1, Concreteness: 0.992), generic and abstract, such as *knowledge* (Specificity: 0, Concreteness: 0.128), but also words that are specific and abstract, such as *neurotic* (Specificity: 0.8, Concreteness: 0.163) and words that are generic and concrete, such as *body* (Specificity: 0.195, Concreteness: 0.994). A substantial, significant negative correlation ($-$0.475, $p < 0.05$) was found between Specificity and Frequency, suggesting, as expected, that very specific words tend to be less frequent in the use. Similarly, we reported a moderate negative correlation between Specificity and Familiarity (Spearman rho: $-$0.228), indicating as expected that more specific words are more likely to be less familiar. This is often the case for specialistic words which score high in Specificity and are perceived to be less familiar to speakers, such as *syphilis* (Specificity: 0.902; Familiarity: 0.193), *scurvy* (Specificity: 0.86; Familiarity: 0.007), and *leprosy* (Specificity: 0.825; Familiarity: 0.076). The opposite situation can be observed for very familiar words which generally tend to be less specific, e.g. *love* (Specificity: 0.179; Familiarity: 0.992), *food* (Specificity: 0.129; Familiarity: 0.984) and *thought* (Specificity: 0.131; Familiarity: 0.969). For Age of Acquisition no significant correlation was observed, suggesting that the age at which a word is acquired is not associated with Specificity, in this study.

In addition, we found that Specificity has no statistically significant correlation with Arousal, but it has a small negative correlation with Valence (Spearman rho: $-$0.173), and a moderate negative correlation with Dominance (Spearman rho: $-$0.216), which are strongly significant. This suggests that more specific words express affective content for which we feel less in control: *tornado* (Specificity: 0.848; Valence: 0.118; Dominance: 0.060), *gangrene* (Specificity: 0.883; Valence: 0.154; Dominance: 0.126), *ulcer* (Specificity: 0.814; Valence: 0.182; Dominance: 0.168) are some examples of words in this context.

Finally, we tested with regression analyses the impact of Specificity on Concretenes and the relation of Specificity with other psycholinguistic variables, comparing linear and quadratic fits. While the relation between Specificity and other psycholinguistic variables is often explained better by a linear function, the emotion-related variables as well as Familiarity and Frequency show a better fit with a quadratic function, suggesting that words with medium levels of Specificity (i.e., basic level lexicon) are more familiar and frequent, as well as less loaded with emotional content, compared to words with very high or very low scores of Specificity.

## Results: How does specificity impact word processing alone, in interaction with concreteness, and when other psycholinguistic variables are controlled?

To investigate the impact of Specificity and other psycholinguistic variables on word processing, we tested the predictive capacity of these variables on Reaction Times in both Lexical Decision and Semantic Decision Tasks. We hypothesized is that Specificity explains a fraction of decision latencies above and beyond Concreteness and other psycholinguistic variables, supporting the idea that Specificity and Concreteness are theoretically distinct variables, and that Specificity plays a role in lexical and semantic access. We expected to see a more prominent role of Specificity in explaining reaction times collected on a semantic task, rather than on a lexical decision task. The nature of lexical decision tasks, in fact, might lead participants to process words at a very shallow level (Pexman et al. 2017). At this level, as suggested by Pexman et al. (2017), the concreteness effect, for instance, may not be captured because participants asked to distinguish words from non-words may not need to process deeply the semantics of words. Conversely, when they are asked to decide whether a word denotes a concrete or an abstract entity, a deep semantic processing is required. At this level, we believe that a Specificity effect may be more easily captured.

We found several psycholinguistic studies of Reaction Times for English words, in which lexical and semantic decision latencies were collected. We selected all of them and verified their coverage with respect to the set of words in our collection. Table 5 summarizes the datasets of reaction times considered for our study, reporting their coverage of the ANEW stimuli (i.e., common words).

**Table 5:** List of datasets of reaction times in lexical decision tasks on English words

Dataset	Task	Codename	ANEW coverage
The British Lexicon Project	LDT	KEULEERS	738 (78.76%)
The English Lexicon Project	LDT	BALOTA	933 (99.57%)
The English Crowdsourcing Project	LDT	MANDERA	937 (100%)
The Massive Auditory Lexical Database	LDT	TUCKER	845 (90.18%)
The Semantic Priming Project	LDT	HUTCHINSON	367 (39.17%)
The Calgary Semantic Decision Project	SDT	PEXMAN	347 (37.03%)

The British Lexicon Project (Keuleers et al. 2012) collects Reaction Times from 78 British students and University employees for about 30K monosyllabic and disyllabic English words and non-words. The English Lexicon Project (Balota et al. 2007) is a standardized dataset of more than 40K words and non-words, for which Reaction Times in a Lexical Decision Task (LDT) have been collected with 816 participants (age mean: 22.86; SD: 6.85) from six Universities in the U.S.A. The English Crowdsourcing Project (Mandera et al. 2020) is a recent dataset containing Reaction Times in a Lexical Decision Task for 62K English words. The data were gathered through an internet vocabulary test completed by over one million participants from the web. The participants in this study have not been instructed to respond as quickly as possible as in classic LDT paradigms, saying whether a string of letter is a word or not. Conversely, participants in this task were asked to say whether they knew the word or not. Participants were expected to give an "I don’t know" response to at least 30% of real words. Data collected in this dataset have a strong correlation (0.75) with the decision latencies collected in the English Lexicon Project. The Massive Auditory Lexical Decision (MALD) Database (Tucker et al. 2019) is a resource that comprises time-aligned stimulus acoustic recording for about 27K words and 10K non-words, along with Reaction Times from Lexical Decision Task completed by 231 monolingual Canadian English student (age mean: 20.11; SD: 2.39). The Semantic Priming Project (Hutchison et al. 2013) is a collection of Speed Naming and Lexical Decision Tasks data for 1.661 target words following related and unrelated primes from 768 subjects (age mean: 21.14; SD: 9.37) recruited across four universities in the U.S.A. The Calgary Semantic Decision Project (Pexman et al. 2017) extends the *megastudy* approaches of word recognition to investigate semantic processing. The researchers collected decision latencies from 321 undergraduate Canadian students (age mean: 21.75; SD: 5.82) for 10K English words using a concrete/abstract Semantic Decision Task, that is deciding whether a word defines a concrete or an abstract entity.

### Specificity as a predictor of decision latencies in lexical and semantic decision tasks

First, we tested the predictive power of Specificity alone over Reaction Times (RT) by performing linear and quadratic Generalized Least Square regressions between Specificity and RTs from all the datasets listed above. In all the cases, the best model in terms of minimization of Bayesian Information Criterion (BIC) is the linear model.

**Table 6:** Results of regression analysis of specificity over RTs (*= $p < 0.05$, **= $p < 0.01$)

RT dataset	$\beta$ value	r-squared
PEXMAN	− 159.359**	0.067
KEULEERS	51.422**	0.049
BALOTA	69.046**	0.038
MANDERA	51.804**	0.036
HUTCHINSON	20.616*	0.012
TUCKER	0.963	2.5e$-$06

Table 6 shows the results of the regression analyses of Specificity predicting decision latencies. Specificity alone explains at most 6.7% of the variance in chronometric data in the PEXMAN dataset (semantic processing latencies), in a negative direction: the more a word is specific, the faster it is processed. However, the number of observations covers only a small portion of the ANEW words (347 stimuli), corresponding to 37% of the total set of words. In KEULEERS, where RTs from British English speakers are collected, Specificity explains about the 5% of the variance in decision latencies. In BALOTA and MANDERA, both covering the whole ANEW dataset, Specificity explains less than the 4% of the variance, about the 1% in HUTCHINSON (less than the 40% of coverage) and a negligible fraction in TUCKER, where Specificity has no significance at all. In all these datasets the direction is positive: the more a word is specific, the more time it takes to process it. We also run models with Concreteness alone, as predictor on the same set of reaction times datasets. We found that Concreteness alone significatively explains decision latencies. The direction of the effect is negative, namely: the more a word is concrete, the faster it is processed. The analyses are stored in the OSF repository.

The fact that LDT and SDT decision latencies tap into different aspects of word processing and require different efforts, is supported by previous literature (e.g., Van Hees et al. 2016; Pexman et al. 2017). For the particular case of Specificity, Lamarra et al. (under review) report a null effect of Specificity in a LDT and significant (negative) effect of Specificity in a SDT, using a set of carefully selected stimuli in Italian, normed with participants’ ratings for Concreteness and Specificity, and balanced across the four conditions for length and frequency. In their study, the authors operationalize the SDT using the same design used by Pexman, namely a semantic decision between concrete and non-concrete (i.e., abstract) concepts. The specificity effect observed in SDT (but not in LDT) partially reflects the pattern of results found in the present study. As a matter of fact, we reported a significant effect of Specificity in SDT (negative direction, namely: more specific shorter decision latencies, less specific longer decision latencies) using Specificity as sole predictor for reaction times as well as in a model where other semantic variables are controlled. Conversely, the impact of Specificity on LDT decision latencies is not consistent across datasets (nor is the effect of other semantic variables).

A notable limitation of our analyses lies in the disproportionate reliance on five distinct datasets of LDT decision latencies compared to only one dataset of SDT decision latencies, a constraint imposed by resource availability in the literature. We advocate for treating the comparison between different LDT datasets as a distinct methodological consideration, supplementary to the juxtaposition of LDT and SDT data. The abundance of LDT datasets affords us the opportunity to elucidate how diverse populations engage with word processing in LDT tasks, which serve as standard metrics of cognitive effort in psycholinguistics and related disciplines. This prompts future investigations to carefully discuss the generalizability of observed effects from participant samples to broader speaker populations.

### Specificity in interaction with concreteness as predictors of reaction times

In this set of analyses, we focused on the interaction between Specificity and Concreteness, hypothesizing that the effect of Concreteness on Reaction Times may depend on the level of Specificity. For this reason, we ran a regression model on Reaction Times, using Concreteness*Specificity as predictors. For this analysis we focused on the datasets with the higher coverage of the stimuli from the ANEW set of words, and for which Specificity proved to have a strong significant impact in the previous analysis, namely KEULEERS, BALOTA and MANDERA, which contain Reaction Times collected during Lexical Decision Tasks. Moreover, we tested our set of variables also on the PEXMAN dataset, that is a collection of Reaction Times in a Semantic Decision Task, despite its low coverage, where Specificity showed a strong significance, too.

**Table 7:** Results of regression analysis of specificity in interaction with concreteness over RTs (**= $p < 0.01$)

RT dataset	PEXMAN	KEULEERS	BALOTA	MANDERA
r-squared	0.286	0.145	0.176	0.137
Specificity $\beta$ value	195.360**	195.412**	238.003**	198.551**
Concreteness $\beta$ value	− 67.940	21.366	− 34.991	14.063
Spec * Conc $\beta$ value	− 283.909**	− 148.186**	− 135.268**	− 149.914**

In the model that considers Specificity, Concreteness and their interaction, reported in Table 7, Specificity continues to have a strong statistical significance when interacting with Concreteness, and a stronger absolute coefficient value, suggesting a stronger effect on reaction times, compared to Concreteness. As a matter of fact, it appears that Specificity drives the significance of the interaction between the two variables, while Concreteness does not have a significant effect on any of the chronometric data collections considered, when Specificity is in the model. In other words, the effect of Concreteness on RTs becomes non-significant when Specificity increases, but that is not true in the opposite case: when Concreteness increases, the effect of Specificity on RTs remains significant. The interaction between the two variables results in significant findings, but with coefficients of opposite sign compared to considering Specificity weights alone. Regarding previous analyses (Table 6), all models show a general improvement in variance explained in the chronometric data, with at least 10 percentage points more.

### Specificity and other psycholinguistic variables as predictors of reaction times

We then investigated how Specificity explains decision latencies along with other psycholinguistic variables commonly acknowledged to be involved in facilitating word processing, namely: Concreteness, Valence, Arousal, Dominance, Familarity, AoA, and Frequency. As in the previous analyses, we only considered datasets of reaction times for which Specificity had a significant impact. Moreover, because each dataset of decision latencies had a different coverage of our data, we checked again for multicollinearity among all variables, for each restricted dataset, before running the regressions. We removed Imageability from all the datasets because it is strongly correlated with Concreteness and it has the highest VIF in all restricted datasets (VIF > 4, as reported in the OSF repository). In addition, we removed Dominance from the PEXMAN analysis because it was strongly correlated with Valence and it showed a problematic VIF value (VIF: 4.774). Table 8 reports the regression results of a model with all the variables on all datasets.

**Table 8:** Regression results on all datasets with all the variables as predictors over RTs (* = $p\,value < 0.05$; ** = $p\,value < 0.01$)

RT dataset	PEXMAN	KEULEERS	BALOTA	MANDERA
r-squared	0.4**	0.493**	0.453**	0.429**
Specificity $\beta$ value	− 104.218*	10.249	33.583**	5.167
Concreteness $\beta$ value	− 113.11**	$-$1.194	$-$18.973*	$-$1.353
Imageability $\beta$ value	–	–	–	–
Valence $\beta$ value	− 30.741	$-$13.612	$-$8.031	$-$2.911
Arousal $\beta$ value	31.893	$-$39.363**	$-$28.647*	$-$26.806**
Dominance $\beta$ value	–	15.663	$-$10.073	$-$10.073
Familiarity $\beta$ value	$-$125.868*	$-$76.238**	$-$60.070**	$-$77.168**
AoA $\beta$ value	153.905**	56.108**	119.139**	67.486**
Frequency $\beta$ value	3.578	$-$90.790**	$-$122.684**	$-$95.123**

As shown in Table 8, Specificity, Concreteness, Valence, and Dominance show no significance in explaining the data in KEULEERS, while Arousal, Familiarity, Age of Acquisition and Frequency are strongly significant ($p < 0.01$). Among these variables, all except Age of Acquisition have a negative effect, meaning that at increasing Reaction Times (slower processing) correspond lower values of Arousal, Familiarity and Frequency. In the BALOTA dataset, Specificity has a strong, positive and significant effect on the Reaction Times, suggesting that higher Specificity leads to longer processing times. Concreteness has a significant negative effect, in line with the Concreteness effect, as well as Arousal, Familiarity, and Frequency (the latter two being strongly significant). The strongest effects in this model are related to Age of Acquisition (positive) and Frequency (negative). Valence and Dominance do not have statistically significant effects on the Reaction Times in the BALOTA collection. All together, these variables can explain the 45% of the variance in decision latencies.

In the MANDERA dataset, neither Specificity nor Concreteness have a significative effect on the Reaction Times. On this dataset, Arousal, Familiarity, Age of Acquisition, and Frequency exhibit consistent behavior with the regression on the BALOTA collection. Dominance and Valence do not show statistically significant effects on these reaction times in LDT. All together, these variables explain 43% of the variance in decision latencies.

Finally, in the PEXMAN dataset, which contains chronometric data collected through a Semantic Decision Task, the behavior of Specificity is the opposite of that observed in the three LDT datasets above, showing a significant negative effect and a notably greater magnitude. The same observations can be applied to Concreteness. Interestingly, the type of semantic decision asked of participants in this task was to determine whether a given word referred to a concrete or an abstract entity. Therefore, one could expect to observe a strong effect of Concreteness because this was the variable of interest in the task. However, Specificity was not part of the task. Additionally, the three emotional variables (Valence, Arousal, and Dominance) and Frequency do not have any significant effect on these data. Familiarity and Age of Acquisition are both significant, with the former showing a highly negative effect and the latter a highly positive and strongly significant one.

Given that the PEXMAN dataset consists of a semantic decision task that assumes stimuli to be pre-labeled as abstract or concrete, we exploited this setting to investigate the interaction between Specificity and Concreteness more deeply. Specifically, we reran the regressions on decision latencies in PEXMAN (dependent variable) for each of the two distinct types of words (abstract and concrete). As independent variables, we used Specificity and added as covariates all other variables, excluding the non-significant ones (Imageability and Dominance, in line with the previous analysis), and Concreteness, which is irrelevant for the type of stimuli, which are already pre-labeled as abstract or concrete. In this way, we observed the impact of Specificity (and all other variables) on the semantic processing times of concrete words, and then of abstract words, to see whether Specificity behaves differently for these two types of words.

**Table 9:** Results of regressions on PEXMAN dataset without concreteness as variable, and with distinction between abstract and concrete words (* = $p\,value < 0.05$; ** = $p\,value < 0.01$)

Dataset: PEXMAN	Abstract (n=181)	Concrete (n=166)	All (n=347)
r-squared	0.11**	0.364**	0.364**
Specificity *β* value	15.133	− 179.415**	− 219.379**
Valence *β* value	− 39.115	10.5	− 31.406
Arousal *β* value	− 26.729	78.805	43.269
Familiarity *β* value	− 174.158*	− 66.833	− 102.328*
AoA *β* value	63.317	276.502**	223.299**
Frequency B *β *value	194.034	− 95.578	− 64.703

As we can see from the results in Table 9, the model performs better in explaining reaction times for concrete words than for abstract ones. Indeed, Familiarity is the only significant predictor of decision latencies for abstract words, with a negative effect (i.e., the more familiar the abstract word, the faster the reaction). Conversely, for concrete words, Specificity and Age of Acquisition are strongly significant predictors of decision latencies.

### Ablation study on most significant variables

Finally, for each of the four collections (KEULEERS, BALOTA, MANDERA, PEXMAN) we conducted an ablation study on all the variables that showed a significant impact on Reaction Times, from which we subtracted the variance explained by all variables minus one, to quantify the fraction of decision latencies explained by each variable, above and beyond all the other variables.

**Table 10:** Individual contribution of each variable above and beyond all other variables, per dataset

Dataset	Variable	R-squared	Delta
KEULEERS	All	0.493**	–
	Familiarity	0.461**	0.032
	Frequency	0.463**	0.030
	AoA	0.469**	0.024
	Arousal	0.476**	0.017
BALOTA	All	0.453**	–
	AoA	0.402**	0.051
	Frequency	0.430**	0.023
	Familiarity	0.444**	0.009
	Specificity	0.449**	0.004
	Arousal	0.449**	0.004
	Concreteness	0.450**	0.003
MANDERA	All	0.429**	–
	AoA	0.402**	0.027
	Familiarity	0.404**	0.025
	Frequency	0.405**	0.024
	Arousal	0.424**	0.005
PEXMAN	All	0.4**	–
	Concreteness	0.364**	0.036
	AoA	0.375**	0.025
	Specificity	0.389**	0.011
	Familiarity	0.389**	0.011

Table 10 reports the ablation study results for each dataset of Reaction Times. We computed multiple regression models by removing each time one of the significative ($p < 0.05$) variables, and we considered the delta between the model with all the variables and the model without the reference variable. According to the ablation, Age of Acquisition is the most important variable in the regressions on LDT collections of decision latencies (with exclusion of KEULEERS, where Familiarity shows the higher impact), and second one in PEXMAN, that is SDT. Removing it from the model on BALOTA leads to a sensible drop in performance (about 5%). The role of Age of Acquisition is important also in PEXMAN, but removing Concreteness has the higher impact on the prediction on these data (about the 4%). Specificity was significant in BALOTA and PEXMAN, but the ablation over this variable does not cause a significant difference in the results on both cases, but we can observe a slightly higher delta on PEXMAN, where the model without this variable registers a 1% difference with respect to a model that comprises it among all the other variables as predictors of reaction times.

### Ad interim discussion

Taking six available datasets of decision latencies collected from different groups of English speakers, we conducted a series of regression models to investigate the role of Specificity (alone and with other variables) in lexical and semantic access. We identified distinct patterns of results across the datasets.

We observed that Specificity has a stronger impact on processing times when the task involves semantic rather than (shallow) lexical judgments. As a matter of fact, in the PEXMAN dataset, when participants were asked to semantically judge whether a word denotes a concrete or abstract entity, the influence of Specificity on processing times was more prominent compared to the other datasets, in which participants were tasked to determine whether a word exists or has been made up. In this Semantic Decision Task, processing time decreased with increasing word specificity: the more specific a word, the faster its processing. This phenomenon is attributed to highly specific words being richly characterized, possessing high-resolution semantic content that facilitates quicker processing times. Conversely, generic words, with low-resolution semantic content, may be processed more slowly as they could refer to various exemplars. Aligned with an exemplar-based theoretical framework of conceptual category representations (e.g., Nosofsky 1986; Van den Bosch and Daelemans 2005; Chandler 2017), participants in a Semantic Decision Task are likely to mentally evoke a referent to discern whether it signifies a concrete or abstract entity. When a word lacks specificity, such as *substance*, participants may mentally generate various potential exemplars within this category before determining its concreteness, potentially leading to extended processing durations. Conversely, encountering a highly specific word like *Aspirin* may prompt participants to readily invoke a single exemplar, resulting in shorter processing durations during the Semantic Decision Task.

The situation differs in Lexical Decision Tasks. Out of the remaining five datasets of decision latencies, only four demonstrated a significant effect of Specificity. Moreover, these effects had the opposite direction compared to the semantic decision dataset. In other words, when participants were asked to judge whether a word is an existing or a made-up one, specific words were processed more slowly than generic words. However, the effect was considerably smaller than in the semantic decision task, as predicted. This reversed pattern of results implies that the two tasks (Lexical and Semantic Decisions) support two distinct cognitive processes: one in which speakers may rely on shallow mechanisms of word recognition (LDT), for which Specificity interferes, albeit to a negligible degree, and the other in which speakers need to access the semantic content of words, for which Specificity facilitates the process.

Moreover, when looking at the interaction between Specificity and Concreteness over decision latencies, we found a significant interaction between the two variables, over both, lexical and semantic decision latencies, but the significance is associated to Specificity, not to Concreteness which, in the model with both variables plus their interaction, results non-significant.

When considering in a comprehensive model various psycholinguistic variables commonly acknowledged to impact decision latencies in lexical and semantic tasks, we found that the contribution of Specificity varies greatly, depending on the dataset. In two out of the four datasets considered (specifically, those for which Specificity alone proved to have an impact over lexical decisions), Specificity does not significantly contribute to explaining the latencies. Interestingly, however, in these two datasets (MANDERA and KEULEERS), Concreteness does not contribute either! This aligns with the idea that Concreteness and Specificity are interrelated variables (Bolognesi and Caselli 2023) and that, in Lexical Decision Tasks, neither of these semantic variables is crucial to distinguish between words and non-words.

Conversely, in one dataset of lexical decision latencies (BALOTA), Specificity significantly contributes, and Concreteness does too, albeit to a lesser extent than Specificity. In this case, the more concrete a word is, the faster it is processed (known as the Concreteness effect), and the more generic a word is, the faster it is processed in determining whether it is a word or a non-word. This suggests the importance of carefully considering Specificity when investigating the so-called Concreteness effect. Neglecting the role of Specificity may lead scholars to erroneously attribute a facilitatory effect solely to Concreteness.

Examining decision latencies in a semantic task (PEXMAN), which proved to be a more suitable task for investigating the role of Specificity and Concreteness, we reported that both variables explain a significant portion of chronometric data, and the effect goes in the same direction: the more concrete and the more specific a word is, the faster its semantic content is processed. To further deepen the understanding of the interaction between Concreteness and Specificity, we ran additional regressions on the semantic decision latencies of two subsets of the PEXMAN dataset: the subset of words labelled as concrete (n=166) and the subset of words labelled as abstract (n=181). Results showed a different pattern on these two types of words. Specificity has a significant effect on the processing times of concrete words (more specific is processed faster than more generic) but this is not the case for abstract words. Here, Specificity does not show a significant effect on processing times, and moreover, the direction of the (non-significant) effect is opposite: abstract generic words are processed slightly faster than abstract specific words. This pattern of results echoes the pattern of results found by Lamarra et al. (under review) on a different set of words, in Italian.

Finally, through an ablation study, we calculated the individual contribution of each variable in explaining decision latencies, above and beyond all other psycholinguistic variables, that is: when all other variables that have an effect over chronometric data are controlled. We reported that, in all datasets, the individual contribution of Specificity and Concreteness is often negligible, compared to other variables such as AoA, Frequency, and Familiarity. The individual contribution of Specificity and Concreteness is also limited in explaining semantic decision latencies (PEXMAN dataset), but it is substantially higher than for lexical decision latencies, as predicted. It should be added that this may be partly due to the peculiarities of the ANEW dataset, which constitutes the pool of words on which chronometric and norming data have been collected. To better appreciate the impact of Specificity (and Concreteness) on decision latencies above and beyond other psycholinguistic variables, it could be more appropriate to use a pool of words where the variation in terms of Concreteness and Specificity is greater.

## Results: Does specificity change across languages and how?

In this final analysis we compared the Specificity ratings collected with the BWS method on English data with the specificity ratings collected with the same method by Bolognesi and Caselli (2023) on the same pool of words (the ANEW dataset) on the Italian translation equivalents. We also compared the other psycholinguistic variables considered in this work between the two languages.

Despite the high correlation between the psycholinguistic variables across English and Italian (average Spearman Rho coefficient: r = 0.744), regressions on chronometric data in the two languages do not show similar behaviors. In Bolognesi and Caselli (2023) Specificity alone explains about the 20% of the variation in LDT chronometric data on Italian (Vergallito et al. 2020), while in our study on English language it explains at most the 5% on LDT in KEULEERS. Regression models that consider also all the other psycholinguistic variable, still show a gap of 7 percentage points between the two languages, with a R-squared of 0.565 on Italian and of 0.493 on English chronometric data. As it can be expected, the correlations between the English and Italian chronometric datasets are low, varying from r = 0.361 (KEULEERS) to r = 0.421 (MANDERA). The PEXMAN dataset, containing data elicited on a different task, reveals an even lower coefficient: r = 0.153. Cross-linguistic correlations between paired datasets of chronometric data are predictably low because translation equivalents may differ in word length, morphosyntactic complexity, phoneme/grapheme frequency or other variables. For this reason, also the discrepancies in the direct comparison between the regression results on reaction times between Italian and English are of limited relevance.

Focusing on norming data, however, and particularly on Specificity, and making some direct word-to-word comparisons across the two languages, we can find some interesting examples. While there exists a robust correlation in Specificity across the two languages (r = 0.758), delving into words where the variable exhibits higher deltas unveils certain cultural biases. The highest difference in terms of Specificity can be observed on the words *mushroom*/*fungo* (EN: 0.781, IT: 0.235, delta: 0.546) and *absurd*/*assurdo* (EN: 0.672, IT: 0.152, delta: 0.520), *rabies*/*rabbia* (EN: 0.839, IT: 0.345, delta: 0.494), and *pasta*/*pasta* (EN: 0.808, IT: 0.325, delta: 0.483), for all of which English values are substantially higher (more specific) than Italian (more generic). Conversely, words that show no substantial difference in terms of Specificity are mostly adjectives or they have an ambiguous word form that can be both nouns or adjectives, such as *victim*/*vittima*, *enraged*/*infuriato*, *loyal*/*leale*, for which the deltas are lower than 0.001.

From a cross-linguistic standpoint, we examined whether the scales employed to operationalize each of the aforementioned variables, including Specificity, were utilized differently by English and Italian speakers. Previous research has indicated a systematic bias among Italians toward assigning higher ratings in Likert scales, when compared to speakers of other European languages, including English (Van Herk et al. 2004).

**Table 11:** Wilcoxon rank sum test results on ratings from Italian and English populations (asterisks for $p\,value < 0.05$)

Variable	Mean EN	Mean IT	W-val	RBC (IT)	CLES (IT)
Valence	0.514	0.515	248952.0	0.019	0.503
Dominance	0.502	0.488	221687.0*	− 0.126	0.472
Frequency	0.641	0.667	179778.0*	0.252	0.546
AoA	0.446	0.405	153013.0*	− 0.298	0.449
Imageability	0.578	0.620	170586.0*	0.227	0.529
Concreteness	0.602	0.551	167676.0*	− 0.341	0.440
Familiarity	0.679	0.622	135845.5*	− 0.384	0.404
Specificity	0.539	0.471	125883.0*	− 0.506	0.410
Arousal	0.453	0.607	34129.5*	0.866	0.751

As shown in Table 11, it is possible to observe language/cultural differences in the use of rating scales from the two populations, but it is not possible to generalize that Italians tend to use higher values. Indeed, with exclusion of Valence, for which the test gave no significance ($p > 0.05$), we can see that for 5 out of 8 variables, that means the 63% of the cases, the English participants used higher values with respect to Italians, as it is explained by negative RBC values.

Finally, we compared the role of specificity in explaining decision latencies during LDTs in English and Italian (for Italian, we used the findings reported in Bolognesi and Caselli (2023)). We observed that specificity has a positive and significant effect on lexical decision latencies in both languages. In other words, in both English and Italian, the more specific a word is, the longer it takes to recognize it. Conversely, the more generic a word is, the faster it is processed in lexical decision tasks. The comparable significance and direction of this specificity effect in the two languages suggest that specificity is a psycholinguistic variable that should be carefully considered in behavioral tasks because it plays a role in lexical access, as evidenced by empirical studies conducted on both English and Italian data.

### Ad interim discussion

In this final exploratory analysis, we compared norming data from various studies that investigated psycholinguistic variables for the ANEW dataset in both English and Italian languages. Our findings revealed overall moderate to high correlation coefficients, indicating a significant cross-linguistic stability across different dimensions of meaning for the analyzed words and their associated concepts.

Notably, Valence exhibited the highest correlation coefficient between English and Italian, suggesting a shared perception of this variable among speakers of different languages and a lesser susceptibility to intercultural variation. One may argue that that the ranking of variables based on correlation coefficients may reflect a continuum from more embodied features (such as valence: a word like *corpse* has a negative valence independently from the language being spoken) to those more dependent on language and specific to culture. For instance, word frequency reflects patterns of word usage, which may vary across languages. Familiarity emerged with the lowest correlation coefficient between the English and Italian datasets, suggesting that familiar entities in English and Italian speaking countries differ.

The variable of primary interest in the current work, Specificity, remains relatively stable between English and Italian, despite qualitative examples illustrating how Specificity can depend on cross-linguistic and cross-cultural variation. We provided a couple of qualitative examples above (*mushroom*, *absurd*, *rabis* and *pasta*), where Specificity varies significantly between the two languages, and words like *victim* and *loyal*, where Specificity is very similar.

*Mushroom* exhibits the highest delta of Specificity between English and Italian, being perceived as much more specific in its English version than in its Italian counterpart (*fungo*). This discrepancy arises from the fact that in Italian, *fungo* encompasses both the spore-bearing organism found in humid forests (like *mushroom* in English) and other types of living organisms like yeasts, which can affect the human body and are referred to as *fungo* in English. In this sense, *mushroom* denotes a specific type of fungus that lacks a translation equivalent in Italian specifically referring to the spore-bearing organism found in forests. Instead, the Italian term *fungo* translates to English with *fungus*, and they arguably share the same level of Specificity. Similarly, *rabis* translates in Italian as *rabbia*, but the Italian word *rabbia* means also *anger*. This may explain why it is perceived to be much more specific in its English than in its Italian version. For words with notable differences between the two languages, such as *pasta* being considerably more specific in English than in Italian, these variations could be attributed to cultural distinctions and categorized as cultural nuances. The vocabulary employed to describe food-related concepts, especially various types of pasta, is arguably more extensive and detailed in Italian compared to English. This linguistic divergence tends to position a term like *pasta* towards the generic end, as opposed to the English language, which may lack a comparable level of specificity in categorizing different types of pasta.

Based on the presented data, the claim posited by Van Herk et al. (2004), according to which Italians tend to use overall higher ratings compared to other linguistic communities, cannot be confirmed: English speakers tend to use overall higher ratings compared to Italian in the 63% of the cases. It should however be clarified that the observations provided by the authors are based on the use of Likert scales, while the data hereby compared has been collected through Best–Worst scaling methods. This suggests that Van Herk et al.’s findings are probably tightly connected to the specific use of Likert scales, and cannot be generalized to scores obtained through other methods, such as for example Best–Worst scaling. Generally speaking, if one were to consider perpetuating the stereotype, the observation of a substantial difference in Arousal suggests a potential distinction between Italians and English speakers in terms of emotional reactivity: for Italian speakers the words in the ANEW dataset are systematically higher in Arousal than for English speakers. English speakers, on the other side, tend to use the highest values of the rating scales when dealing with Concreteness and Specificity, while Italians show higher sensitivity to Imageability.

Finally, as noted above, the populations providing ratings in the datasets utilized for the cross-linguistic correlation analysis lack control for sociodemographic variability. Our data collection on Specificity primarily involved British, but also American and Canadian English adult speakers (mean age: 42.39, SD: 14.44), with approximately 90% of them not enrolled in any university program, while a prevalent pattern in many rating studies involves participants predominantly being students enrolled in bachelor’s degree programs (mostly from psychology classes), speakers of one specific variety of English. The experiences and lexical skills characterizing different types of speakers are likely to play a role in cross-linguistic comparisons, which future research shall consider.

## General discussion

The primary objectives of the current set of studies were threefold: first, to present a dataset of Specificity judgments collected through the Best–Worst scaling method based on English data; second, to conduct a comprehensive analysis of the relationship between Specificity and Concreteness, along with other psycholinguistic variables frequently implicated in the exploration of conceptual abstraction and in relation to decision latencies, within the context of English data; and third, to delve into and discuss potential cross-linguistic distinctions between English and Italian ratings of Specificity.

In essence, our analyses aim to contribute to the broader understanding of how Specificity and Concreteness, along with other psycholinguistic variables, relate to one another under the general mechanisms of abstraction, which involves both, semantic categorization and the difference between concrete and abstract concepts. These two mechanisms are then discussed in relation to word processing.

To summarize and interpret our results, we first reported a positive correlation between the specificity ratings obtained through the BWS method and those automatically extracted from WordNet, although the correlation is not strong. This outcome was anticipated, as WordNet encompasses encyclopedic knowledge that speakers are unlikely to have mastered in such detail. This discovery underscores the significance of the specificity ratings dataset presented and released herein. It highlights the need for future researchers to recognize that word specificity, as extracted from a knowledge base like WordNet, may slightly differ from that elicited by speakers. Depending on the focus and research question addressed, it might be more theoretically sound to use speakers’ generated judgments of word specificity rather than specificity scores automatically extracted from WordNet.

We then investigated the role of Specificity in explaining decision latencies in lexical tasks (word recognition) and semantic tasks (determining whether a word denotes a concrete or an abstract entity). Specificity alone has a stronger effect on semantic tasks than on shallow lexical tasks, as expected. The direction of the effect is negative in the semantic decision task (the more specific are the words, the faster they are processed), while it is positive in the lexical decision tasks (the more specific are the words, the slower they are processed), and it is in line with Iliev and Axelrod (2017). When considering the interaction between Specificity and Concreteness in explaining decision latencies, the interaction is significant, and it seems to be driven by Specificity. Moreover, it seems that the Specificity effect is observable on concrete words mainly, while it is non-significant among abstract words. This finding is particularly important because the so-called concreteness effect, widely acknowledged in the scientific literature, is typically supported by empirical data (behavioral and neuroscientific studies) that use stimuli not balanced for Specificity, due to the general lack of resources that can be used to operationalize and measure this variable.

Since we reported a positive correlation between Specificity and Concreteness, and we reported that Specificity explains decision latencies and drives the interaction with Concreteness in explaining decision latencies, in line with Bolognesi and Caselli (2023), we argue that it could be the case that concreteness effects reported in the literature may need to be readdressed, as it may actually be explained by Specificity or by an interaction between the two variables, and not by Concreteness alone. In other words, it could be the case that concrete and abstract stimuli used to investigate the concreteness effect are actually specific-concrete words and generic-abstract words. This lack of control over Specificity may lead to erroneously labeling the observed effect in chronometric data.

A note of caution should be added. The present study is conducted on a pool of words (the ANEW dataset and its Italian version) for which there are no specifics on the rationale that motivated stimuli selection. The dataset contains ratings on dimensions of emotional content for many English words. This pool of words has been used in several subsequent norming studies aimed at collecting human judgments on other psycholinguistic variables and correlating these with emotional norms (e.g., Vergallito et al. 2020; Montefinese et al. 2019). Words in the ANEW dataset, therefore, are not systematically balanced for Concreteness and Specificity. Future research aimed at investigating the role of Specificity (and Concreteness) in explaining decision latencies in word processing may focus on carefully constructed datasets of stimuli containing highly specific and highly generic words. A preliminary study (Lamarra et al. under review) worked in this direction, using a dataset of Italian words carefully balanced also for length and frequency, and reported no effects of Specificity (nor Concreteness) on lexical decision latencies, and a negative effect of both Concreteness and Specificity on semantic decision latencies (more concrete and more specific: faster processing), with no interaction between the two variables, suggesting that each of the two variables explains an individual portion of decision latencies.

Finally, we compared the Specificity ratings presented herein for English data to those previously collected for Italian data (Bolognesi and Caselli 2023) on the same pool of words through qualitative observations. The correlation between the two languages is on average medium-high on all the psycholinguistic variables, despite the words in each being expressed differently. The lowest correlations are observed for Frequency, suggesting that word usage differ across languages; Arousal, suggesting that the intensity of a word emotional content is perceived differently across languages; and Familiarity, suggesting that familiar and non-familiar words differ across languages. For our primary variable of interest, Specificity, we found a medium-high correlation between English and Italian ratings. Nevertheless, we also reported that there are words that display a high delta of Specificity between the two languages, and these may be attributed to linguistic phenomena, such as the higher polysemy of a term in one language than in another, or to the peculiarities of the semantic field to which the word belongs: some semantic fields are likely to be lexically richer, in one language than in another, and this may result in translation equivalents being evaluated differently in terms of their Specificity.

## Conclusion

In recent years, the study of conceptual abstraction has witnessed a renewed surge of enthusiasm and scholarly attention. This renewed wave of interest can be attributed, in part, to new lexical resources made available and heightened interest in the phenomenon of generalizations performed by the human mind and from the perspective of artificial intelligence. The core theoretical contribution of this paper is that it is crucial to avoid conflating Concreteness and Specificity within the study of abstraction in human and artificial minds. Failure to distinguish between these two variables could impede a comprehensive understanding and appreciation of their respective contributions to linguistic behavior.

## Footnotes

1 The scripts can be downloaded from: http://www.saifmohammad.com/WebPages/BestWorst.html.

2 https://www.prolific.com/.

3 https://www.qualtrics.com/.
